# The impact of acute violent videogame exposure on neurocognitive markers of empathic concern

**DOI:** 10.1093/scan/nsae031

**Published:** 2024-05-10

**Authors:** Mary B Ritchie, Shannon A H Compton, Lindsay D Oliver, Elizabeth Finger, Richard W J Neufeld, Derek G V Mitchell

**Affiliations:** Graduate Program in Clinical Science and Psychopathology, Department of Psychology, University of Western Ontario, London, ON N6A 3K7, Canada; Brain and Mind Institute, University of Western Ontario, London, ON N6A 3K7, Canada; Brain and Mind Institute, University of Western Ontario, London, ON N6A 3K7, Canada; Graduate Program in Neuroscience, Schulich School of Medicine and Dentistry, University of Western Ontario, London, ON N6A 3K7, Canada; Campbell Family Mental Health Research Institute, Centre for Addiction and Mental Health, Toronto, ON M5T 1R8, Canada; Department of Psychiatry, University of Toronto, Toronto, Ontario M5R0A3, Canada; Robarts Institute, University of Western Ontario, London, ON N6A 3K7, Canada; Department of Clinical Neurological Sciences, The University of Western Ontario, London, ON N6A 3K7, Canada; Lawson Health Research Institute, London, ON N6C 2R5, Canada; Parkwood Institute, St. Joseph’s Health Care, London, ON N6C 0A7, Canada; Graduate Program in Neuroscience, Schulich School of Medicine and Dentistry, University of Western Ontario, London, ON N6A 3K7, Canada; Department of Psychiatry, Schulich School of Medicine and Dentistry, University of Western Ontario, London, ON N6A 3K7, Canada; Department of Psychology, Faculty of Social Science, University of Western Ontario, London, ON N6A 3K7, Canada; Brain and Mind Institute, University of Western Ontario, London, ON N6A 3K7, Canada; Department of Psychiatry, Schulich School of Medicine and Dentistry, University of Western Ontario, London, ON N6A 3K7, Canada; Department of Psychology, Faculty of Social Science, University of Western Ontario, London, ON N6A 3K7, Canada; Department of Anatomy and Cell Biology, Schulich School of Medicine and Dentistry, University of Western Ontario, London, ON N6A 3K7, Canada

**Keywords:** violent video game, emotional empathy, fMRI, violent gaming, neurocognitive markers

## Abstract

Research examining the purported association between violent gaming and aggression remains controversial due to concerns related to methodology, unclear neurocognitive mechanisms, and the failure to adequately consider the role of individual differences in susceptibility. To help address these concerns, we used fMRI and an emotional empathy task to examine whether acute and cumulative violent gaming exposure were associated with abnormalities in emotional empathy as a function of trait-empathy. Emotional empathy was targeted given its involvement in regulating not only aggression, but also other important social functions such as compassion and prosocial behaviour. We hypothesized that violent gaming exposure increases the risk of aberrant social behaviour by altering the aversive value of distress cues. Contrary to expectations, neither behavioural ratings nor empathy-related brain activity varied as a function of violent gaming exposure. Notably, however, activation patterns in somatosensory and motor cortices reflected an interaction between violent gaming exposure and trait empathy. Thus, our results are inconsistent with a straightforward relationship between violent gaming exposure and reduced empathy. Furthermore, they highlight the importance of considering both individual differences in susceptibility and other aspects of cognition related to social functioning to best inform public concern regarding safe gaming practices.

Videogames are a popular consumer good, with 2.5 billion gamers spending almost $2 billion worldwide in 2022 (Newzoo, 2022). As videogames continue to increase in popularity, so too have concerns regarding the potential impact of gaming on social functioning (particularly aggression). An estimated 85% of videogames now contain some form of violence (American Psychological Association, Task Force on Violent Media, 2020), suggesting that the 97% of teens (Kühn *et al*., 2019) and 60% of young adults (18–29; Brown, 2017) who play videogames will encounter violent content. Many of these individuals spend a significant portion of their time engaging with videogames, where 13% of game-playing teens report gaming for at least 3 hours a day (Lenhart *et al.*, 2008). Given the level of exposure to and involvement in virtual violent actions, concern regarding the potential interplay between violent videogames and real-world aggression is understandable.

The impact of violent videogames on aggressive behaviour continues to be a topic of debate (Anderson *et al*., 2010; Bushman and Anderson, 2023; Ferguson, 2007; Mathur and VanderWeele, 2019; all are reviews or meta-analyses). Though some continue to doubt the purported effects of violent gaming on aggression (Devilly *et al.*, 2023), others suggest that pooled estimates in different meta-analyses suggest small but significant effects of violent gaming on aggressive behaviour (Mathur and VanderWeele, 2019). Although meta-analyses often report a small to moderate association between violent videogames and aggression (*r* = 0.08 to 0.19; APA, 2020), the interpretation of these effects has varied. While some identify violent videogames as a risk factor for subsequent aggression (Möller and Krahé, 2009; Coker *et al*., 2015; Groves and Anderson, 2019; Shao and Wang, 2019; APA, 2020), others suggest the effect is trivial (see meta-analysis by Ferguson *et al*., 2020). The validity of significant findings has been called into question due to methodological concerns (Bushman and Anderson, 2023; Gallar and Ferguson, 2020; Mathur and VanderWeele, 2019; all reviews or meta-analyses). For example, the extant literature has been criticized for its use of correlational designs and overreliance on experimental proxy paradigms (i.e. lab measures of aggression, self-report) that may have limited generalizability to real-world behaviours (Elson and Ferguson, 2014; Ferguson, 2015; Gallar and Ferguson, 2020; Markey, 2015; Ritter and Eslea, 2005; all reviews). Lab measures of aggression have also been criticized for producing demand effects, rather than real aggression (e.g. Tedeschi and Quigley, 1996, 2000). Bushman and Anderson (2023) note that future studies should make attempts to reduce potential demand effects through suspicion checks or the use of overt measures. Critics have also commented that many studies fail to control key extraneous variables such as individual differences in trait anger or exposure to family violence (Ferguson *et al*., 2008a). Bushman and Anderson (2023), however, suggest that it may be inappropriate to statistically control all possible extraneous variables, as in many cases, researchers may be parsing out variance from their own dependant variable. Instead, Bushman and Anderson (2023) suggest characterizing extraneous variables as moderators in the association between violent gaming and aberrant social outcomes.

Notably, the restricted focus on aggressive outcomes may result in a tendency to overlook the potential impact of violent videogames on other critical, though less frequently measured, aspects of social functioning. Emotional empathy (i.e. the process in which one shares or shows concern for another’s emotional experience, evoking altruistic motivation; e.g. Smith, 2006) is one such factor thought to facilitate prosocial behaviour and inhibit antisocial behaviour across the lifespan (e.g. Blair, 2001; Jolliffe and Farrington, 2004; Decety *et al*., 2016; Weisz and Zaki, 2018). Importantly, emotional empathy can be understood from at least partially dissociable (Nezlek *et al*., 2007) state and trait perspectives (Shen, 2010; Reniers *et al*., 2011). While trait empathy is an enduring disposition with well-established links to pathological patterns of antisocial behaviour (e.g. Hare, 1996; Blair, 2005), the extent to which one responds empathically can vary based on contextual factors (e.g. Nezlek *et al*., 2007). Singer and Lamm (2009), e.g. describe empathy as a malleable and highly flexible phenomenon. In line with this perspective, extraneous factors that have been shown to modulate an individual’s empathic state include positive or negative daily affect (Nezlek *et al*., 2001), interpersonal closeness (Batson *et al.*, 2002; Cialdini et al., 1997; Simpson *et al*., 1995; Lyu *et al*., 2022), length of interaction (Powell and Roberts, 2017), and the size of the interaction group (Davis *et al*., 1996). Others suggest that motivation to respond empathically or avoid empathic responding can vary as a function of the empathizer’s characteristics as well as situational factors (Cikara *et al*., 2014; Zaki, 2014). According to these theories, in certain intergroup contexts, it may be possible for those who are typically harm averse to overcome this aversion and engage in collective violence to promote group survival (e.g. Cikara *et al*., 2014). Though violent gaming exposure has been linked to affective change (e.g. increased state hostility; Anderson, 1997; Anderson and Bushman, 2001), very little is known about the acute impact of violent gaming on state emotional empathy at a neurocognitive level.

Much of the existing work concerning violent gaming and empathy is correlational in nature and tends to focus on the association between trait empathy and violent gaming history rather than state empathy. From this perspective, trait empathy (empathic concern) has been shown to partially mediate the association between violent videogame use and lower levels of prosocial behaviour (Fraser *et al.*, 2012). That is, violent videogame use appears to be associated with lower prosocial behaviour through a mechanism of decreased trait emotional empathy, particularly towards strangers. Some studies highlight the association between violent gaming and components of social cognition such as the ability to accurately identify emotional facial expressions in others (Kirsh *et al*., 2006; Diaz *et al*., 2016). Specifically, violent gaming has been associated with reduced recognition of happy and disgusted facial expressions, but increased recognition of fearful facial expressions (Kirsh *et al*., 2006; Diaz *et al*., 2016). Given that empathic responding can be influenced by situational factors (Nezlek *et al*., 2007), coupled with the broad implications of empathy for social functioning (Hoffman, 2000; Blair, 2005; Smith, 2006), it is important to consider the impact of violent gaming on emotional empathy through experimental procedures.

Although models of aggression in the context of violent gaming note the importance of biological factors in addition to social, cognitive, and developmental ones (e.g. DeWall *et al*., 2011), the potential neural mechanisms remain unclear. Evidence from neuroimaging studies identify a network of brain regions associated with states of emotional empathy. Meta-analyses of such studies highlight activation in areas including the anterior insula, anterior cingulate cortex (ACC), inferior frontal gyrus (IFG), dorsomedial prefrontal cortex (dmPFC), amygdala, and the inferior parietal lobule during tasks designed to elicit emotional empathy (for reviews or meta-analyses, see Bzdok *et al*., 2012; Kogler *et al*., 2020; Lamm and Singer, 2010; Singer and Lamm, 2009; for more recent and original work, please see Sadeghi *et al*., 2022; Zhao *et al*., 2021). It should be noted that many of these regions have been implicated in multiple facets of empathy, including cognitive empathy (i.e. perspective-taking; see meta-analysis by Kogler *et al*., 2020). Nevertheless, lesion studies have helped clarify the relative importance of some of these neural regions; these studies suggest that IFG and anterior insula play a critical role in emotional empathy, but not in cognitive empathy (Hillis, 2014; Shamay-Tsoory, 2015; both reviews). Thus, if acute violent gaming exposure has a negative impact on processes related to emotional empathy, it is reasonable to expect that activity in these regions to stimuli shown to induce empathic responding will be impacted.

Though sample sizes are small (*n* < 15), some studies showed evidence of abnormalities in emotion-related brain regions (e.g. dACC, left insula) during gameplay among gamers versus non-gamers (Weber *et al*., 2006; Gentile *et al*., 2016). Others have found that acute exposure to violent videogames disrupts activity in areas of the prefrontal cortex associated with cognitive control (Wang *et al*., 2009) and response inhibition (Hummer *et al*., 2010). Similar effects are observed with post-gaming electroencephalographic data, where reduced cingulate cortex, limbic, and temporal area activation has been recorded during a social inclusion; suggesting reductions in emotional engagement during social processing (Lai *et al*., 2019). Still, some have found no evidence that violent gameplay modulates emotion-related neural processes (Szycik *et al*., 2017; Kühn *et al*., 2018) or resting state activity (Pan *et al*., 2018). Importantly, there has also been an emerging study by Lengersdorff *et al*. (2023) that suggests, using Bayesian statistics, that there is no impact of exposure to violent gaming on empathy for pain. Notably, these studies examined brain activation during gameplay or following acute exposure and did not account for potential cumulative effects of years of violent gaming. This may be important given the evidence that time spent engaging with violent videogames may be a better predictor of social functioning than acute exposure (Weber *et al*., 2006). For example, Compton *et al*. (2022) reported that reduced activity in parts of an action simulation network implicated in empathy was associated with cumulative but not acute violent videogame exposure. Other studies have revealed evidence that abnormal prefrontal cortex activation during cognitive inhibition has been associated with both acute and cumulative violent gameplay (i.e. 2 weeks; Hummer *et al*., 2010, 2019). There has also been mixed evidence as to whether frequency of violent gameplay affects neural desensitization to painful images or violent stimuli. While some studies provide evidence of neural desensitization among habitual gamers (Staude-Müller *et al*., 2008; DeWall *et al*., 2011; Miedzobrodzka *et al*., 2022), others report no differences in pain perception between gamers and non-gamers (Gao *et al*., 2017; Goodson *et al*., 2021). As the empirical picture is mixed, additional research is needed to clarify the acute and cumulative impact of violent gameplay on broader aspects of social functioning.

## Current study

The violent videogame literature has predominantly relied on correlational designs with a focus on aggressive outcomes. Despite evidence of an association between violent gaming and aggressive behaviour (for meta-analytic support, please see Anderson *et al*., 2010), the methodological approach taken in these studies has been criticized and the validity of reported effects remains controversial (for a critical review, please see Ferguson, 2020), and questions remain about the potential neurocognitive mechanism involved and role of individual differences (e.g. Ferguson *et al*., 2008a). To help overcome some of these concerns, the current study examined whether acute and cumulative violent videogame exposure was associated with abnormalities in neurocognitive markers of emotional empathy, and whether any observed effects varied as a function of trait-empathy. This approach allowed us to assess the impact of acute violent gameplay and gaming history on factors not only implicated in regulating aggressive behaviour, but also other socially desirable characteristics (e.g. compassion, respect for others’ feelings) that are not necessarily captured by lab measures of aggression. Further, by incorporating an experimental manipulation, this approach allowed us to examine the impact of violent gaming on state-based changes in social functioning more broadly.

Our central hypothesis was that acute violent gaming exposure would increase the risk for aberrant social behaviours by altering the aversive value of distress cues. The current study examined one particular correlate of aberrant social behaviour, i.e. the function of neural regions implicated in empathic responding. Specifically, we predicted that emotional empathy-related blood-oxygen-level-dependant (BOLD) signal changes in the amygdala, anterior insula, left IFG, and aspects of superior temporal cortex would be significantly reduced in those exposed to violent versus nonviolent gaming (specifically during the presentation of negatively valanced images). We also predicted that, as individuals with lower trait empathy (e.g. high coldhearted traits) are at risk for higher rates of aggression (Ritchie *et al*., 2022) and reduced responsiveness to distress cues (e.g. Fraser *et al*., 2012), these individuals may be particularly susceptible to such effects of violent gaming.

## Methods

### Participants

Fifty-two healthy, right-handed adults (20 males, 32 females) with a mean age of 23 (range 18–30, SD = 3.9) were recruited by word of mouth and through fliers posted across a Canadian university and surrounding community. Flyers included general lab posters for the Emotional Cognition Lab, as well as posters citing an fMRI study involving videogame play. Those interested were asked to email the lab and were told that the study would involve an MRI, playing a videogame, and completing a series of computer tasks. All participants self-reported being in good physical health and did not meet criteria for any psychiatric disorders as determined by the Structured Clinical Interview for DSM-5—Research Version (SCID-RV; First *et al.*, 2015). Written consent was obtained, and compensation was provided. This study was approved by the Office of Human Research Ethics at University of Western Ontario, London, Ontario, Canada. Notably, three participants were unable to complete the full study due to technical difficulties (*n *= 1) or discomfort during the MRI scan (*n *= 2). An additional three participants were removed from the final analyses due to a low response rate (<60%) during the emotional empathy fMRI task. The final sample therefore included 46 adults (16 males, 30 females) with a mean age of 23 (SD = 3.9).

In the current study, a series of repeated measures ANOVAs and bivariate correlational analyses were conducted. As noted by Compton *et al*. (2022), sample sizes were calculated *a priori* to satisfy these analyses based on the effect size estimates from previous work (ranging from medium to large; Fecteau *et al*., 2008; Bushman and Anderson, 2009). Specifically, for our group contrasts, we referenced effect sizes from prior work involving the impact of violent video games on helping behaviours (Bushman and Anderson, 2009) and neuroimaging studies comparing activation in empathy-related brain areas during violent and nonviolent video gameplay (Gentile *et al*., 2016). For our correlational analyses, we also drew upon a neuroimaging study from our group that examined interactions between coldheartedness and responsivity of relevant brain areas to affective stimuli (Vieira *et al.*, 2017), and a study that examined the interaction between coldheartedness and action-simulation-related responsivity (Fecteau *et al*., 2008). Extrapolating from these studies, an estimated minimum effect size of *r* = 0.43 (*d* = 0.953) for our primary analyses, given the target samples size of *N *= 40 and an *n *= 20 for our between group cells, yields a power in excess of 0.85 in each case.

### Procedure

The current study took place over two visits. During the first visit, formally trained researchers conducted the SCID-RV to rule out any psychiatric disorders, as well as the Wechsler Abbreviated Scale of Intelligence (WASI-11; Wechsler, 2011) to assess general cognitive function. Participants also completed a series of media use and trait questionnaire measures. Media use questionnaires (i.e. Media Preference Scale for film and television and the Video Game Questionnaire; Anderson and Dill, 2000) were used to ensure similar baseline levels of media consumption across conditions and to explore the association between gaming history (i.e. in recent months, and across adolescents and early adulthood) and outcome measures. Trait measures included the Psychopathic Personality Inventory-Revised (PPI-R; Lilienfeld and Widows, 2005) to assess coldhearted traits, the Autism Quotient (AQ; Baron-Cohen *et al.*, 2001; Spielberger, 1999). The AQ was used as a control variable to ensure groups did not differ as a function of autistic traits, as there is evidence to suggest that autism spectrum disorder is associated with an increased risk of problematic gaming behaviours (e.g. Craig *et al*., 2021). Similarly, the STAXI-2 was also included to ensure groups did not differ as a function of pre-existing levels of aggression. We selected the measures used in the current study, as they demonstrate good psychometric properties and are commonly cited throughout the literature. For example, the AQ is one of the most commonly cited measures of autism to date (Lundqvist and Lindner, 2017). Further, there is evidence that, in healthy adults, callous traits as measured by the PPI-R are associated with reduced distress cue awareness (Oliver *et al.*, 2015), reduced empathic concern (but intact cognitive empathy; Oliver *et al.*, 2016), and reduced activity in emotion-related brain areas to fearful cues (Han *et al.*, 2012). Descriptive statistics and a breakdown of recent media consumption across gaming group are presented in Table 1. Notably, our data suggest that selection effects were not a concern, as our sample’s violent videogame frequency ranged from 0 hours to 171 hours in recent months. Further, 26% of our sample had played less than 10 hours of violent gaming in recent months while only 13% of our sample had played over 100 hours of violent gameplay in recent months.

**Table 1.** Descriptive statistics for questionnaire measures

	Violent condition (*n* = 22)			Nonviolent condition (*n* = 24)			T-test
Measure/Task	*M*	SD	Range	*M*	SD	Range	*P-value*
Age	23.82	3.86	18–29	22.17	3.70	18–30	.15
Biological sex	Male *n* = 7; Female *n* = 15			Male *n *= 9; Female *n* = 15			–
IQ	115.77	11.97	92–131	115.72	10.54	92–133	.49
Coldhearted traits	49.73	13.71	25–81	45.50	11.24	30–79	.13
Autistic traits	108.45	10.26	90–130	107.42	14.06	86–138	.39
Trait anger	21.40	11.13	1–36	16.67	12.07	6–36	.09
Film & television (hours per month)	–	–	–	–	–	–	–
Recent	5.64	7.75	0–35	5.04	6.65	0–34	.78
High School	6.03	7.59	0–34	5.60	4.61	0–18	.82
Cumulative video game use	–	–	–	–	–	–	–
Recent	55.82	45.98	0–164	48.78	50.54	0–171	.63
High School	150.73	135.0	0–506	136.25	139.37	0–583	.73

*Note: M *= mean; Coldhearted Traits = T-score derived from PPI-R normative sample.

(Lilienfeld & Widows, 2005); Autistic Traits = Autism Quotient (Baron-Cohen, 2001); Trait Anger = State-Trait.

Anger Expression Inventory (STAXI-2; Spielberger, 1999); Film & Television = Media Preferences Scale (Anderson & Dill, 2000); Cumulative VGE = Cumulative Videogame Exposure via the Video Game Questionnaire (Anderson & Dill, 2000); IQ = Intelligence Quotient via the Wechsler Abbreviated Scale of Intelligence (WASI-11; Wechsler, 2011).

During the second visit (∼1 week later), participants were randomly assigned to play either a violent (*n *= 22) or nonviolent (*n *= 24) version of Grand Theft Auto-V (GTA-V) for 1 hour prior to completing a series of tasks while undergoing fMRI. Those in the violent group engaged in a series of missions involving heists, shoot outs, torture, street theft, carjacking, and arson that included depictions of blood and dead bodies. Those in the nonviolent group played a modified version of GTA-5 devoid of violent graphics (e.g. the environment did not include weapons, blood, screaming, or dead bodies) and actions (e.g. participants could not injure non-player characters, steal, or cause damage). Instead, participants engaged in a series of missions within the same virtual environment that included racing, piloting airplanes, and skydiving (see supplementary materials for information about the missions).

After an hour of gameplay, participants entered the MRI scanner. While in the MRI scanner, participants watched two pre-recorded videos designed to stimulate video game live streaming (an activity growing in popularity where someone else plays video games live online; El Afi *et al.*, 2021). The two videos (21.5 and 22.5 minutes, respectively) were congruent with each participant’s assigned gaming group and showed a pair of hands using the same controller the participants used in the bottom corner of the screen. Gameplay videos were used to maintain the effects of videogame exposure throughout the study and reduce the likelihood of extinction while in the scanner. Participants underwent fMRI during the first video and anatomical and diffusion tensor imaging (DTI) scans were recorded during the second video. Interweaved between the gameplay videos, participants completed two counterbalanced fMRI tasks, including an emotional empathy task and an action simulation task. The current study focuses on the results of the emotional empathy task, as the results of the action simulation task have been published elsewhere (Compton *et al*., 2022). Task responses were recorded using a Current Designs button box and sound was delivered by Sensimetrics in-ear ambient sound-attenuating headphones. Stimuli for each task, as well as the pre-filmed gameplay, were presented electronically using E-Prime 3.0 software (Psychology Software Tools, Pittsburgh, PA).

### Emotional empathy task

**Fig. 1.**
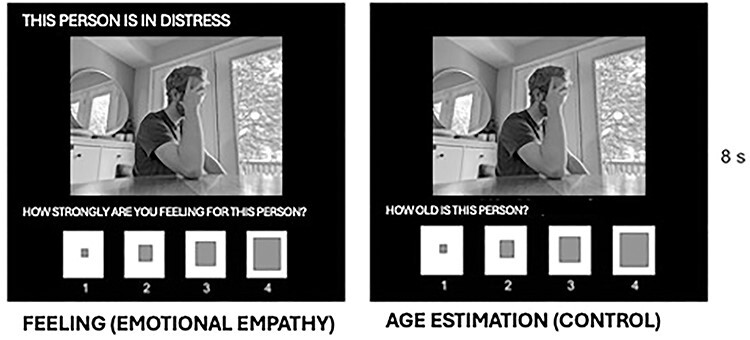
Trial structure of the emotional empathy task (adapted from Dziobek *et al*., 2011; Oliver *et al*., 2018).

Note. For copyright purposes, this image is a representation of stimuli presented during the emotional empathy task and was not part of the formal experiment.

The current study used an adapted version of the Multifaceted Empathy Test (MET; Dziobek *et al*., 2011) and was identical to one previously used in our group (see Figure 1; Oliver *et al*., 2018). Unlike the MET, which includes indices of cognitive and emotional empathy, this adaptation focused exclusively on emotional empathy and control conditions. In the emotional empathy condition (i.e. feeling), participants were presented with social, emotionally charged images and were asked to rate how strongly they feel for the people in the image on a scale from 1 (not at all) to 4 (very strongly). To minimize the need for cognitive empathy, each image included a tagline identifying the emotional state of the people in the image. In the control condition (i.e. age), participants are presented with the same images and asked to identify the age of the person in the image on a scale from 1 (very young) to 4 (very old).

The emotional empathy task included 36 images (18 positive and 18 negative) from the MET (Dziobek *et al*., 2008) and the International Affective Picture System (IAPS; Lang *et al.*, 2008). All 36 images were displayed within both the feeling and age conditions, for a total of 72 images displayed. Images were equally divided and randomized across three runs (i.e. 24 images per run). Within each run, participants completed a series of 4 counterbalanced blocks separated by an 18-second inter-block interval. Within each of the four counterbalanced blocks, two blocks presented the feeling condition (i.e. six images per block) and two blocks presented the age condition (i.e. six images per block). Each run contained an equal number of positive and negative images (i.e. 12 positive and 12 negative images). Negative and positive images were presented to measure empathy related to negative affect (distress) and positive affect, respectively. The valence of images within each block was randomized. Table 2 provides a breakdown of the images per run.

**Table 2.** Breakdown of images per run

	Run 1		Run 2		Run 3		
Task condition	Positive images	Negative images	Positive images	Negative images	Positive images	Negative images	Total images
Feeling	6	6	6	6	6	6	36
Age	6	6	6	6	6	6	36

**Fig. 2.**
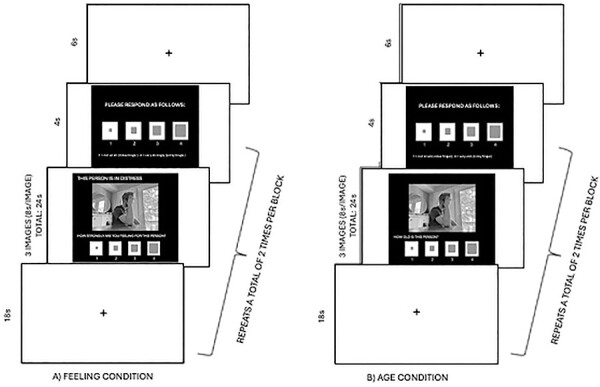
Trial structure of a block from the emotional empathy task (adapted from Dziobek *et al*., 2011; Oliver *et al*., 2018); (a) feeling condition; (b) age condition.

During each block, participants were shown a fixation for 6 seconds, followed by a 4-second instruction slide. A series of three randomized images of the same condition (i.e. feeling or age) were then shown for 8 seconds each (24 seconds total). These images were followed by another six second fixation, and three additional randomized images from the same condition (i.e. feeling or age) were then shown for 8 seconds each (24 seconds total). For both conditions, participants were instructed to respond to each image during the 8-second interval. These images were then followed by an 18-second inter-block interval to allow the BOLD signal to return to baseline before proceeding to the next block. This process was then repeated three additional times to complete a run. Figure 2 depicts the ending sequence of each block. Each run lasted 6.6 minutes and the total task duration was 19.8 minutes. Notably, the Evoked Potential Operant Conditioning System (EPOCs) of interest were obtained during the 8 seconds in which each image was displayed.

### MRI data acquisition

Participants were scanned in a single session using a 3 Tesla Siemens Prisma scanner with a 32-channel head coil at Western University’s Robarts Research Institute. fMRI images were taken with a T2*-gradient echo planar imaging sequence with whole-brain coverage [repetition time (TR): 1250 ms; echo time (TE): 30 ms; field of view (FOV): 192 mm; 96 × 96 matrix; multi-band acceleration factor of 3 and a grappa acceleration factor of 2]. For functional scans, 57 contiguous slices of 2 × 2 mm in-plane with a slice thickness of 2 mm (2 × 2 × 2 mm voxels) were obtained. While participants were watching the second pre-filmed video of gameplay, approximately in the middle of the scanning session, a high-resolution, T1 weighted, anatomical scan was obtained with whole-brain coverage (TR: 2300 ms; TE: 2.98 ms; FOV: 256 mm; 256 × 240 matrix; 192 axial slices; 1 × 1 × 1 mm voxels).

### Analyses

#### fMRI pre-processing

Analysis of Functional NeuroImages (AFNI; Cox, 1996) software was used to conduct analyses at the individual and group level. To correct for motion across time, volumes from each task were registered to a functional volume adjacent to the anatomical scan. Individual volumes were excluded if a motion sensor limit of 0.3 mm was surpassed from one TR relative to the previous TR (average TRs lost: 1.2%). The remaining volumes were spatially smoothed using an isotropic 4-mm Gaussian kernel. Time series data were then normalized, where each voxel was represented as a per cent change from the mean voxel intensity. Regressors were created for each of the task conditions (i.e. feeling and age) by convolving the blocked stimulus events with a gamma-variate hemodynamic response function. The in-block instructions for each task condition were modelled as regressors of no interest. Motion regressors, including six de-meaned motion parameters, were also included in the model. The BOLD response was then fitted to each of these regressors so that linear regression modelling could be conducted for all participants across conditions. For each regressor, this created a beta coefficient and t-statistic for each voxel. All data were transformed into standard Talairach and Tournoux space to allow for group level analyses.

#### Region of Interest analysis

The following paragraphs describe a series of pre-planned analyses, unless otherwise defined as exploratory. An independent functionally defined Region of Interest (ROI) corresponding to an area implicated in emotional empathy (left IFG) was derived from a previous neuroimaging study conducted by our lab involving a sample of healthy adults (Oliver *et al*., 2018). An area implicated in both emotional and cognitive empathy was also derived from the same study (superior temporal cortex). Using Oliver *et al*. (2018) original dataset, each region was defined by the feeling > age contrast (revealing emotional empathy-related activation) at *P* < .00001 and *P* < .0001, respectively. Additional anatomically defined ROIs were extracted from AFNI’s TT_Daemon atlas, including the left and right anterior insula and amygdala. All masks were resampled to ensure alignment with the current dataset.

For each ROI, the per cent signal change from baseline to the emotional empathy and age estimation conditions was extracted. These values were then entered into a series of 2 (between-subjects condition: violent versus nonviolent video game exposure) × 2 (task: feeling versus age estimation) × 2 (valence: positive versus negative stimuli) factorial repeated measures ANOVAs. Exploratory bivariate correlations were conducted with the sample split across gaming group to examine the relationship between emotional empathy-related activity (i.e. feeling—age difference score) in significant ROIs and coldheartedness scores as measured by the PPI-R. Given recent evidence that dissociable patterns of neural activity can be associated with acute versus cumulative violent gaming exposure (Compton *et al*., 2022), additional exploratory correlational analyses were conducted collapsed across gaming group. Specifically, a series of bivariate correlations were performed to examine the relationship between emotional empathy-related activity in significant ROIs and cumulative gaming exposure in recent months. Recent cumulative violent videogame exposure (i.e. in recent months) was derived from the Videogame Questionnaire calculated using a method initially adopted by Anderson and Dill [2000; (violent content + violent images) × frequency in recent months], where higher scores indicate a greater frequency of gameplay and a higher degree of violent content. Datapoints falling ± 3 SD from the group mean were identified as univariate outliers and omitted from the corresponding analysis. Mahalanobis distance was calculated to identify bivariate outliers in the association between ROI signal change and covariates of interest (i.e. coldheartedness, cumulative gaming). Notably, we do not feel this approach was too conservative, as we lost a maximum of 1 or 2 datapoints across analyses. The Benjamini-Hochberg procedure was used to correct multiple comparisons within each ANOVA (*n *= 6) and across all correlational analysis (*n *= 26). All effects reported as significant survived correction.

Exploratory Bayesian analyses were performed on non-significant main effects of gaming group (i.e. violent versus nonviolent) to determine the probability that a null hypothesis was true [i.e. emotional empathy-related activity (feeling—age) did not differ across gaming group in each ROI]. One benefit of Bayesian statistics is that they allow you to make inferences about the null hypothesis (Quintana and Williams, 2018). For each ROI, a Bayesian independent samples t-test was conducted in SPSS to draw inferences about the difference between gaming groups. Bayesian Pearson correlations were also conducted to examine the association between feeling ratings and cumulative gaming history. The Bayes Factor (i.e. the likelihood that the null hypothesis is true divided by the likelihood that the alternative hypothesis is true) was estimated using a non-informative prior. Bayes Factors above 1 support the null hypothesis, while Bayes Factors below 1 support that the alternative hypothesis is true (Lee and Wagenmakers, 2014; Quintana and Williams, 2018). Importantly, Bayes Factors greater than 3 are thought to reflect ‘substantial evidence’ while Bayes Factors above 10 are thought to reflect strong evidence for the null hypothesis (Jeffreys, 1961; Kelter, 2021). Notably, to avoid being overly conservative in determining what was and was not supportive of the null, we did not correct for multiple comparisons within the Bayesian analyses.

#### Whole brain analysis

A series of exploratory independent samples t-tests were conducted with the whole-brain functional data using AFNI’s 3dttest++ command to identify any emotional empathy-related activation (feeling > age) that differed significantly across the violent and nonviolent gaming groups. Additional exploratory whole-brain group contrasts were conducted separately with trait coldheartedness and cumulative gaming as a covariate to identify neural regions that interact with gaming group across valence not captured by the ROI analysis. The resultant slopes of the two gaming groups were compared. Notably, one participant was identified as a bivariate outlier across every cluster identified when coldheartedness was included as a covariate (Mahalanobis distances ranged from 14 to 36; *P *< .001). As such, the whole brain group contrasts with trait coldheartedness as a covariate were repeated with this participant excluded. The results of this analysis are presented below. Whole brain contrasts were corrected for multiple comparisons, *P* < .05, using 10 000 Monte Carlo simulations. Based on this analysis, clusters should be larger than 19 continuous voxels to be considered significant.

## Results

### Behavioural analysis with task performance

Mean feeling ratings (i.e. ratings of how strongly participants felt for each image) were calculated for positive and negative feeling trials across gaming group. A 2 (valence: negative versus positive) × 2 (gaming group: violent versus nonviolent) repeated measures ANOVA was conducted to examine the impact of gaming group on mean feeling ratings. There was no main effect of valence (*F* (1) = 0.22, *P *= .64), gaming group (*F* (1) = 0.46, *P *= .50), or interaction identified (*F* (1) = 0.93, *P *= .34), suggesting mean feeling ratings did not differ as a function of gaming group or stimulus valence. Notably, Bayesian independent samples t-tests provided support for the null hypothesis that emotional empathy ratings for both negative (Bayes Factor = 2.71) and positive (Bayes Factor = 4.52) affect did not differ across gaming group.

#### Correlations

A series of bivariate correlations were conducted across gaming group to examine the association between coldhearted traits and emotional empathy ratings on the MET after acute exposure to the violent or nonviolent game. In the nonviolent gaming group, coldhearted traits were associated with lower feeling ratings towards positive stimuli (*r *= –0.45, *P =* .03), while no association was found among negative stimuli (*r *= –0.16, *P* = .46). In the violent gaming group, no associations were found among coldhearted traits and feeling ratings towards positive (*r *= –0.10, *P *= .66) or negative (*r *= –0.39, *P *= .08) stimuli. Collapsing across gaming group, additional bivariate correlations were conducted to examine the association between MET emotional empathy ratings and recent cumulative gaming exposure. No significant associations were identified across negative (*r *= 0.11, *P *= .47) or positive (*r *= 0.05 *P* = .75) stimuli. Bayesian Pearson correlations provided support for the null hypothesis that recent cumulative gaming exposure was not associated with feeling ratings towards positive (Bayes Factor = 8.26) or negative stimuli (Bayes Factor = 6.68).

### Region of Interest analysis

**Fig. 3.**
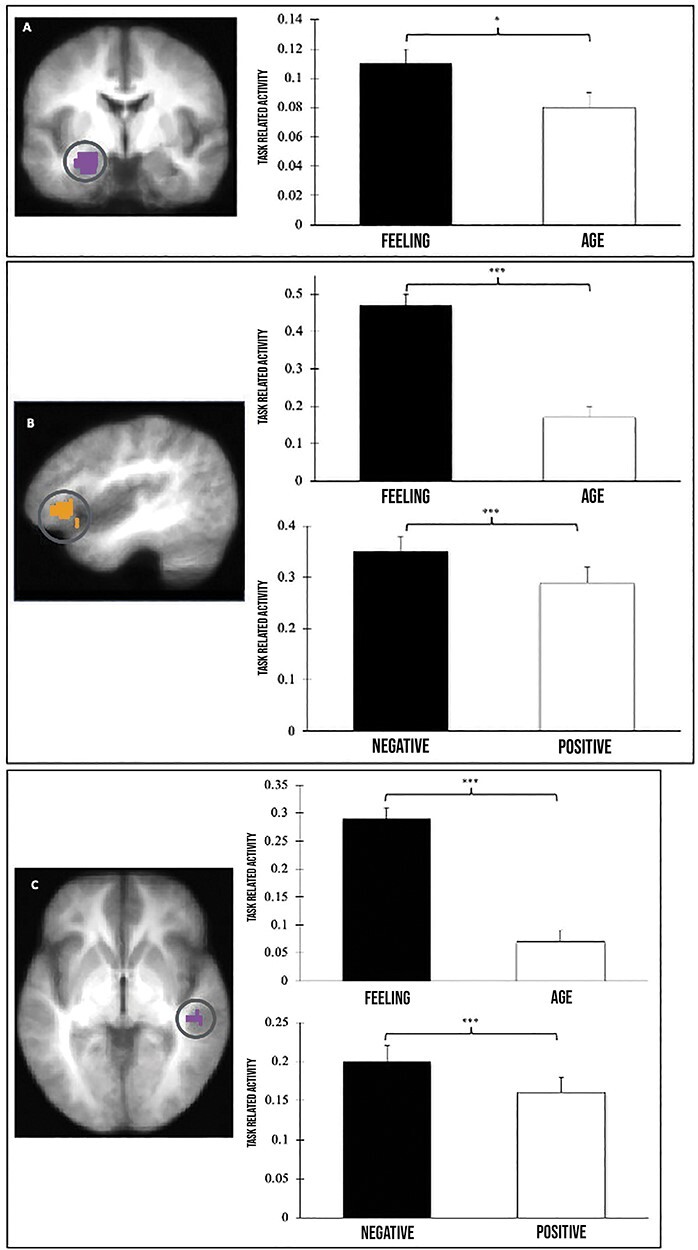
Significant regions of interest. (a) Anatomically defined left amygdala and a graph depicting the main effect of task condition (*P *< .05). (b) Functionally defined left inferior frontal gyrus region of interest derived from Oliver *et al*. (2018) feeling > age contrast (*P *= .00001) and the main effect of task and valence in this region. (c) Functionally defined region of interest measuring the overlap between emotional and cognitive empathy (i.e. an area of superior temporal cortex spanning the frontal pole and superior temporal suculus posteriorly to include the temporoparietal junction) derived from Oliver *et al*. (2018) feeling > age contrast (*P *= .0001) and the main effect of task and valence in this region.

Note. * *P *< .05; *** *P *< .001.

A series of factorial repeated measures ANOVAs were conducted to examine the impact of videogame exposure on per cent signal change in ROIs previously associated with emotional empathy (Oliver *et al*., 2018). *A priori* ROIs included functionally defined areas within left inferior frontal gyrus (IFG), superior temporal sulcus (STS) and temporoparietal junction (TPJ), as well as an anatomically defined region in anterior insula and amygdala. For each ROI, the per cent signal change related to the feeling and age estimation tasks was calculated and entered into a series of 2 (between-subjects condition: violent versus nonviolent video game exposure) × 2 (task: feeling versus age estimation) × 2 (valence: positive versus negative stimuli) factorial repeated measures ANOVAs. Significant results from the ROI analyses are graphically presented in Figure 3.

In the left amygdala, there was a significant main effect of task (feeling > age; *F* (1) = 4.60, *P *< .05); however, there was no main effect of valence (*F *= 4.09, *P *= .05) or gaming group (violent > nonviolent; *F *= 0.003, *P *= .96). In the left IFG, there was a main effect of task (feeling > age; *F* (1) = 80.00, *P *< .001) and valence (negative > positive; *F* (1) = 14.27, *P *< .001); however, there was no main effect of gaming group (*F* (1) = 3.89, *P *= .06). In an area of superior temporal cortex, there was a significant main effect of task (feeling > age; *F* (1) = 84.29, *P *< .001) and valence (negative > positive; *F* (1) = 12.49, *P *< .001), but no main effect of gaming (*F* (1) = 2.01, *P *= .16). No main effects were found across the remaining ROIs, including the right amygdala (*F*TASK (1) *=* 0.11, *P *= .75; *F*VALENCE (1) *=* 0.40, *P *= .53; *F*GAMING (1) *=* 0.17, *P *= .68), left anterior insula (*F*TASK (1) *=* 0.38, *P *= .54; *F*VALENCE (1) *=* 0.01, *P *= .98; *F*GAMING (1) *=* 2.24, *P *= .14), or right anterior insula (*F*TASK (1) *=* 0.56, *P *= .46; *F*VALENCE (1) *=* 1.04, *P *= .31; *F*GAMING (1) *=* 0.60, *P *= .44). No significant interactions were identified across analyses (*P *> .10; see Supplemental Materials). Notably, Bayesian independent samples t-tests provided support for the null hypothesis that emotional empathy-related activation (i.e. feeling—age difference score) towards negative and positive images did not differ across gaming group across all ROIs (Table 3).

**Table 3.** Bayesian inferences for non-significant effects of gaming group (i.e. violent versus nonviolent) within regions of interest

	Bayes factor for gaming group differences	
ROI	Negative stimulus valence	Positive stimulus valence
Left Amygdala	3.70	3.25
Right Amygdala	3.82	2.73
Left Anterior Insula	4.16	4.28
Right Anterior Insula	4.42	2.38
Left IFG	4.40	4.48
Right STS/TPJ	4.35	2.13

*Note*: All Bayes Factors provide support for the null hypothesis; Bayes Factors above 1 provide support the null.

Hypothesis, while Bayes Factors below 1 support that the alternative hypothesis as true (Lee and Wagenmakers, 2014; Quintana and Williams, 2018).

#### Correlations

A series of bivariate correlations were then conducted to test the prediction that the relationship between coldheartedness and reductions in activity in these ROIs would be greater among those exposed to violent versus nonviolent gaming. Correlations were conducted with the sample split across gaming group to examine the relationship between coldheartedness scores as measured by the PPI-R and emotional empathy-related activity (i.e. feeling—age difference score) towards negative and positive images in the left amygdala, left IFG, and an area of the superior temporal cortex. No significant correlations were identified (*P *> .10). Additional bivariate correlations were conducted collapsed across gaming group to examine the association between emotional empathy-related activity in significant ROIs and recent cumulative gaming exposure. No significant correlations were identified and Bayesian Pearson correlations provided support for the null hypothesis (Bayes Factors ranging from 4.01 to 8.32).

### Whole brain analysis

Whole-brain group contrasts revealed no significant differences between gaming groups in emotional empathy-related activation (feeling > age) for negative and positive affect (separately; *P *> .10). Additional t-tests were conducted with coldhearted traits as a covariate to determine whether the association between gaming group and emotional empathy (defined by the contrast, feeling—age) for negative and positive affect varied as a function of coldhearted traits. Notably, a bivariate outlier was identified between coldhearted traits and empathy-related activation for negative and positive affect. This participant was removed from whole brain analyses where coldhearted traits were a covariate to ensure the results were not inordinately skewed (Malhanobis Distance = 14.3 to 34.6 across initial clusters).

**Fig. 4.**
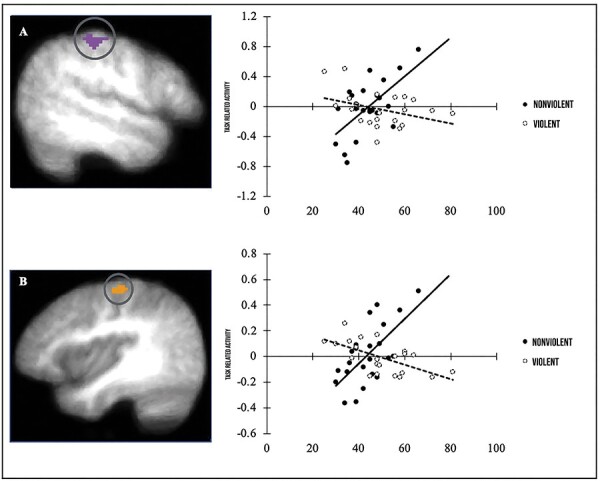
Significant whole brain clusters.

Note. Solid line = nonviolent gaming group; broken line = violent gaming group.(a) Cluster within the left somatosensory cortex; (b) cluster within the right motor cortex.

The covariate analyses revealed no significant clusters for empathy towards negative affect, suggesting that coldhearted traits did not interact with violent gaming exposure to modulate neural activity for negative affect (*P *> .10). However, when examining activity elicited by the empathy for positive stimuli condition, two significant clusters within the left somatosensory cortex and right motor cortex were identified (see Figure 4). For the cluster within somatosensory cortex, there was a significant positive association between activation and coldhearted traits within the nonviolent group (*r *= 0.63, *P *< .001). That is, higher coldhearted traits were associated with enhanced activity in somatosensory cortex during positive empathy trials. However, no significant effect in this region was observed in the violent group (*r = *–0.36, *P *> .10). The slope of these correlation coefficients was compared using the Fisher r-to-z transformation for two independent samples, which confirmed that the slope of the lines did differ significantly (*z *= –3.54, *P *< .001).

In an area within the right motor cortex, similar contrasting associations were identified. Within the nonviolent condition, higher coldhearted traits were associated with enhanced activity in the right pre- and postcentral gyrus (*r *= 0.66, *P *< .001) towards positive empathy trials. Conversely, in the violent condition, coldhearted traits were inversely associated with activation for empathy towards positive affect in an area of the right motor cortex (*r *= –0.62, *P *< .01). Fisher r-to-z transformation revealed a significant difference in the slope of these coefficients (*z *= –4.80, *P *< .001).

Additional exploratory whole brain fMRI analyses were conducted to examine the effect of recent cumulative violent gaming exposure [i.e. (violent content + violent images) × frequency in recent months] on emotional empathy-related activity. For each valence (i.e. negative and positive stimuli) a paired samples t-test was conducted in AFNI comparing emotional empathy-related activity (feeling—age) with cumulative violent gaming exposure as a covariate of interest, collapsed across gaming group. No significant clusters were identified across valence (*P *> .10). To ensure that there were no unanticipated interactions involving gaming group, these analyses were repeated in an exploratory fashion within each gaming group. Again, no significant clusters were identified (*P *> .10).

## Discussion

The current study sought to examine whether acute and cumulative violent videogame exposure was associated with abnormalities in neurocognitive markers of state-based emotional empathy, and whether any observed effects varied as a function of trait-empathy. The methodological approach taken in the current study was felt to address many of the violent gaming research considerations recently highlighted by Anderson and Bushman (Anderson and Bushman, 2023), including the need to view extraneous variables as moderators as well as the need to use less overt measures of aggression to reduce possible demand effects. As expected, empathic states were associated with increased activity in areas implicated in emotional empathy, including the amygdala, left IFG, and superior temporal cortices, thereby replicating prior effects observed using the same task (Oliver *et al*., 2018). However, contrary to our predictions, we did not find evidence that acute violent videogame exposure resulted in emotional empathy-related BOLD signal changes in the amygdala, anterior insular, left IFG, and aspects of the superior temporal cortex. Similarly, there was no evidence of group differences through whole brain analyses or in relation to cumulative gaming exposure. Bayes analyses provided support for all null hypotheses. However, trait-based differences were observed as a function of gaming condition during whole brain analyses for empathy towards positive stimuli within the left somatosensory and right motor cortex. This effect was unexpected, and additional evidence is needed before reliable conclusions can be drawn. Nevertheless, the effect may represent preliminary evidence for an asymmetry in susceptibility to the effects of violent gaming as a function of trait empathy.

### Impact of acute and cumulative violent gaming on broader social functions

The violent gaming literature has traditionally focused on aggression as an outcome despite concerns that lab-based and self-report measures of aggression may have limited generalizability to real-world behaviours (Ritter and Eslea, 2005; Elson and Ferguson, 2014; Markey, 2015; Gallar and Ferguson, 2020). For example, lab measures of aggression have failed to correlate with rates of violent crime and some tests of frontal lobe function implicated in aggressive behaviour (e.g. Ferguson *et al*., 2008b; Ferguson and Rueda, 2009). Others suggest that lab measures of aggression may reflect compliance to perceived experimenter demands rather than actual aggressive tendencies (e.g. see Tedeschi and Quigley, 1996, 2000 for a review). To overcome these concerns, researchers have begun to explore the association between violent gaming history and broader aspects of social functioning such as empathy and emotion recognition (e.g. Diaz *et al*., 2016; Fraser *et al*., 2012; Lengersdorff *et al*., 2023), which are skills commonly linked to the modulation of aggressive or other antisocial outcomes (Blair, 2001; Decety *et al*., 2016; Jolliffe and Farrington, 2004; all reviews or meta-analyses). In some cases, measuring reliable constructs linked to aggression rather than aggression itself may represent a form of ‘non-deceptive obfuscation’ that reduces the risk of experimenter demand effects (e.g., Zizzo, 2010) and elucidates the impact of violent gaming on broader aspects of social functioning.

While much of this research focuses on trait-based constructs via correlational methods, there is a subset of literature linking violent gaming exposure to changes in state-based affect (e.g. increased state hostility; e.g. Anderson and Bushman, 2001). Empathy is one such construct (commonly associated with prosocial behaviour, emotional well-being, and social connectedness, and inversely associated with aggression; Cohen, 2004; Morelli *et al*., 2015, 2017) that can vary moment-to-moment due to extraneous factors (e.g. interpersonal closeness; Nezlek *et al*., 2007; Batson *et al*., 2002). Though trait-empathy has been identified as a potential mediator in the association between violent gaming and aggressive outcomes (e.g. Diaz *et al*., 2016; Fraser *et al*., 2012), the current study considered how acute exposure to violent gaming impacts state-empathy, and whether these effects vary as a function of trait empathy. Here, we found no evidence that acute exposure to violent gaming disrupted state-based emotional empathy self-report ratings at a behavioural level or regional BOLD signal on a neural level. This finding was consistent with studies suggesting violent gameplay does not modulate emotion-related neural processes (e.g. Szycik *et al*., 2017; Kühn *et al*., 2018) or resting state activity (Pan *et al*., 2018). Importantly, this finding was also consistent with findings from an emerging study suggesting no impact of exposure to violent gaming on empathy for pain (Lengersdorf and colleagues, 2024). Notably, while Compton *et al*. (2022) reported similarly null results when examining the impact of acute violent videogame exposure on activity within the action simulation network (e.g. mirror empathy; Iacoboni *et al*., 1999), significant disruptions in the left IFG were observed in relation to participants’ cumulative gaming history.

Though these findings highlight that time spent engaging with violent videogames may be a better predictor of social functioning than acute exposure alone, we did not observe an effect of cumulative gaming exposure on state-based emotional empathy at a neural level. One possible explanation for this null result is that the measure of emotional empathy used in the current study (much like previous studies using measures of aggression) was still susceptible to experimenter demand effects. In the Compton *et al*. (2022) study, mirror empathy was covertly measured using a task in which participants were presented with a series of images or videos of a hand pressing buttons on a button box and were asked to either observe or execute an identical button press in response. In contrast, the current study utilized an empathy task in which participants provided feeling ratings for images captioned with a statement acknowledging how the person in the image was feeling. This is a validated paradigm (e.g. Dziobek *et al*., 2011) that has yielded effects in clinical and basic populations (e.g. Autism, Borderline Personality, Narcissism; Dziobek *et al*., 2008, 2011; Ritter *et al*., 2011) evoking consistent responses in specific neural regions (e.g. ACC, IFG, dmPFC). Despite the reliability and validity of this measure, it may be more susceptible to participant bias than the mirror empathy task in which the purpose of the task is less clear to participants. This discrepancy highlights the potential importance of considering more indirect methods of examining constructs within experimental paradigms.

### Individual differences in susceptibility

In the past, the violent gaming literature has been criticized for failing to consider or control for the impact of extraneous variables such as individual differences on observed effects (Ferguson *et al*., 2008a). From a trait perspective, empathy scores have been shown to partially mediate the association between violent gaming and reductions in prosocial behaviour (e.g. Diaz *et al*., 2016; Fraser *et al*., 2012). The current study adds to this literature by considering the influence of coldhearted traits (an individual difference factor associated with reduced responsiveness to distress cues; e.g. Blair, 2001) on one’s susceptibility to the purported effects of violent gaming. Notable trait-based differences were observed through whole brain analyses for empathy towards positive stimuli within the left somatosensory and right motor cortex. In both regions, higher coldhearted traits were associated with greater BOLD signal activity within the nonviolent gaming group. Among the violent gaming group, higher coldhearted traits were associated with reduced BOLD signal activity in an area of the right motor cortex, but not in the somatosensory cortex.

These findings were not predicted *a priori*, and the effects should be interpreted with caution without replication. While we cannot rule out unanticipated reaction time or other motor-related outcomes, the motor responses were matched across condition to reduce the likelihood that our results could be explained by motor-related effects. Further, we would not anticipate coldhearted traits to be related to general reaction time processes. It is interesting to note, however, that the somatosensory and motor cortices are activated during the observation of emotional expressions, and are considered fundamental in the process of emotional mirroring (e.g. Adolphs *et al*., 2000; Preston and de Waal, 2002; Kropf *et al*., 2019; Sun *et al*., 2022). Furthermore, they are thought to represent neural markers for emotional empathy towards negative (e.g. pain; Singer *et al*., 2004) and positive (e.g. happy; Jabbi *et al*., 2007) stimuli. Some studies have suggested that youth or adults with psychopathic tendencies (e.g. higher coldhearted traits) tend to show typical or heightened activation in the somatosensory cortex relative to control participants when shown images of others in pain (e.g. Decety *et al*., 2009, 2013; Marsh *et al*., 2013). One interpretation of the current results is that participants higher on coldhearted traits may be more sensitive or attuned to positive stimuli, and that this initial response is attenuated with exposure to violent content. Interestingly, there are data to suggest that the motivational salience of rewarding stimuli and reward seeking more generally is either intact (Blair *et al*., 2004) or even enhanced in individuals with high coldhearted traits (Cleckley, 1988; Scerbo *et al*., 1990; Bjork *et al*., 2012). However, it should be noted that these findings relate to positive outcomes for the self rather than others. While an interesting possibility, this interpretation remains highly speculative and further work is required to replicate and elucidate these effects. For example, tasks such as the Implicit Association Test (IAT; Greenwald et al., 1998, have been used to indirectly measure empathy and attitudes towards empathy by detecting subconscious associations or biases between concepts (e.g. race or violence) and outcomes (e.g. good versus bad; Patané *et al*., 2020). Such tasks have been shown to overcome the limitations of direct or self-report measures of empathy (e.g. Kämpfe *et al*., 2009) and have revealed that violent gaming is associated with increased endorsement of implicit, but not explicit, criminal attitudes following exposure to Grand Theft Auto gameplay (Shaw *et al*., 2014).

### Limitations and future directions

To assess the wider implications of violent gaming use beyond aggression, the current study targeted state-empathy, an aspect of social cognition that is associated with prosocial behaviours, and inversely associated with aggressive behaviour. In contrast to a prior study reporting an effect of cumulative gaming exposure on aspects of motor empathy (Compton *et al*., 2022), here we did not observe such effects on emotional empathy. While the current experiment may have still been susceptible to demand characteristics, the former was likely less susceptible (Compton *et al*., 2022). As such, future studies may consider using less overt measures within experimental paradigms (e.g. mirror empathy task, psychophysiological/electromyography measures).

Another potential limitation concerns the focus on a specific style of video game. In the current study, participants engaged in gameplay from a third-person perspective, in which they were able to see the body of the character they were controlling. It is possible that this perspective created a barrier between the participant and the gaming character that would not be present from a first-person perspective (e.g. Skyrim, Phasmophobia). As well, effects may differ as a function of the salience of distress cues within various games or media types (e.g. film, TV). For example, in the violent game used here, opportunities to view distress cues among non-playable characters (NPCs) depend on the storyline or mission played, and may be less overt than distress cues presented in violent film or TV or through virtual reality-based games.

With recent criticisms of sample size in violent gaming research (Bushman and Anderson, 2023), it is worth acknowledging the potential for our study to be underpowered in detecting effects. Further, at the time of our sample planning, Bushman and Anderson’s (2023) paper had not been published. Notably, in this paper, Bushman and Anderson cite an estimated effect size of *r *= 0.20. The effect size used to estimate the sample size needed in the current study was based on previous literature suggesting an *r* of 0.43 (e.g. Bushman and Anderson, 2009; Gentile *et al*., 2016). Future research may consider using this more conservative estimate to determine sample size. Importantly, an emerging study with higher power (*n *= 89; Lengersdorff *et al*., 2023) reported a similarly null effect. While our power analysis suggested sufficient power in excess of 0.85 for our primary analyses with a sample size of *N* = 40 (*n* = 20 per group), it remains possible that the study was underpowered, particularly in detecting trait effects. As such, the analyses concerning possible trait effects should be considered preliminary and potential interactions with traits cannot be ruled out based on the current sample. We have, however, demonstrated in our previous published work (Oliver *et al*., 2016) that empathy ratings on the Emotional Empathy task relate to individual differences in coldheartedness among typically developing individuals (Oliver *et al*., 2016), as well as those with neurodegenerative conditions affecting empathy (Oliver *et al.*, 2014). Further, brain activation in regions considered critical for emotional empathy (see Shamay-Tsoory, 2015 for a review) correlate with behavioural ratings on the same task used in this study (Oliver *et al*., 2018). We agree that caution should be used in interpreting effects presented in the current study; however, a strength of the current study is that it utilizes a paradigm with demonstrable sensitivity at a neural and behavioural level. Further, behavioural performance on the task (ratings) correlate with brain activity in our critical ROIs. The correlation between motional empathy ratings and ROIs suggests our task is capable of detecting meaningful intersubject variations. Notably, our task was based on the Multifaced Empathy Task which demonstrates empirical utility and sensitivity throughout the literature (Dziobek *et al.*, 2008), but incorporates a cognitive empathy component in which we were not interested in assessing during the current study.

The current study may be limited by the use of a convenience sample (i.e. university students). Though the vast majority of university students report playing videogames (70%; Jones, 2003), expanding the participant pool into the community would allow for a more diverse range of participants in terms of prior gaming experience, increased generalizability, and a greater range of trait-based scores and social contexts. Given our recruitment strategy, one concern may be that we oversampled from individuals with high rates of gaming. However, there was significant variance; our sample’s violent videogame frequency ranged from 0 hours to 171 hours in recent months (SD = 46.79). Further, 26% of our sample had played less than 10 hours of violent gaming in recent months while only 13% of our sample had played over 100 hours of violent gameplay in recent months. Notably, while university samples may produce a smaller range of coldhearted trait scores than clinical samples, there is evidence to suggest that the prevalence of psychopathy within university samples may be greater than that of the general community (Boduszek *et al.*, 2021; Sanz-García et al., 2021). While recent meta-analyses suggest that the effects of violent gaming are observed across gender (Prescott et al., 2018), there is some research to suggest that the magnitude of the effect may be larger among males (e.g. Bartholow and Anderson, 2002). Given that the convenience sample collected in the current study was predominantly females, it would be beneficial to conduct studies like the current study with a larger sample balanced between males and females to account for potential gender-based effects. Notably, when looking at the overall sample (i.e. collapsed across gaming group), there were no significant gender differences observed in the ROIs (*F* = 0.10 to 2.22, *P *< .05). Further, the PPI-R is a measure of coldheartedness that focuses on personalogical features (Morey, 2003), which is a preferred option when assessing broader psychopathic traits within non-offending community samples. In the current study, a sufficient range of scores were observed in the current study (i.e. collapsed across gaming group, coldhearted trait scores ranged from 25 to 81).

Notably, a measure of coldhearted traits was included in the current study as these broader affective features have been shown to better predict aggressive behaviour than traditional measures of empathy (see meta-analysis by Ritchie *et al*., 2022). While deficits in empathy are a component of coldhearted traits, other socially relevant affective characteristics are also accounted for, including a lack of guilt, manipulation, and unemotional characteristics of shallow affect (Frick *et al.*, 2014; Frick and Ray, 2015). To better understand the unique impact of empathic traits, future studies may wish to incorporate more traditional measures of empathy (e.g. Interpersonal Reactivity Index; Davis, 1983). Future research may also wish to explore susceptibility as a function of developmental level (e.g. see Kirsh, 2003 for a review) as well as life experience (e.g. exposure to trauma; see Couette *et al*., 2020 for a review), as these factors have been shown to interact with aspects of social cognition. It is worth noting that there are alternative measures of psychopathy available within the literature. Many of these scales, including the Levenson Self-Report Psychopathy Scale (Christian and Sellbom, 2016), Self-Report Psychopathy Scale-IV (Paulhus *et al.*, 2014), and Triarchic Psychopathy Measure (Patrick, 2010), consider broader facets of psychopathy beyond coldhearted traits that may be interesting to considered as an individual difference factor in future studies.

Finally, it is important to acknowledge that the emotional empathy task utilized in the current study did not distinguish between affective sharing and empathic concern despite evidence in the literature of a distinction between affective sharing (e.g. ability to share the emotional experience of another) and empathic concern (e.g. feelings of sympathy and compassion that evoke altruistic motivation to care for the welfare of another; Batson *et al.*, 1987, 1991). However, given logistical difficulties in assessing each given the constraints of *f*MRI and temporal resolution, attempting to separate them would require significantly longer task duration, stimulus repetitions, and a more complex task. It would also require the assumption that affective sharing would not also trigger empathic concern even if it was not mentioned in the instructions. Furthermore, it is unclear whether separate neural targets exist that would uniquely account for affective sharing and empathic concern. For these reasons, we followed prior work (Oliver *et al*., 2018) in using the instruction ‘FEEL FOR’ to capture aspects of both empathic concern and affective sharing in line with previous work. We acknowledge, however, that should the opportunity arise to distinguish empathic concern from affective sharing at a neural level, it would have a valuable application to violent gaming research and elsewhere.

## Conclusions

The current study contributes to the growing literature examining the effects of violent videogame exposure on aspects of social functioning (i.e. state-empathy) by considering the impact of acute and cumulative gaming exposure as well as trait-based differences in susceptibility to purported effects. While we found no evidence that acute or cumulative gaming exposure influences BOLD signal activity in regions associated with state-based emotional empathy, notable interactions with gaming and empathic traits emerged in the left somatosensory and right motor cortex. Specifically, the significant association between coldhearted traits and positive empathy-related activation in the somatosensory and motor cortex observed in the nonviolent group was inverted for the violent group. Further research is needed with ‘non-deceptive’ covert measures of multiple aspects of social cognition, in more diverse samples, and using immersive games to better determine any potential effects of gaming. Given the increasing popularity (Newzoo, 2022), violence (APA, 2020), and immersive nature of videogames (Newzoo, 2022), it is important to continue to objectively evaluate the potential impact of gaming exposure on aspects of social functioning across development to best inform public concern regarding safe gaming practices.

## Supplementary Material

nsae031_Supp

## Data Availability

The data underlying this article will be shared on reasonable request to the corresponding author.
