# Supplementary material for: The impact of acute violent videogame exposure on neurocognitive markers of empathic concern
Source: Soc Cogn Affect Neurosci. 2024 May 10;19(1):nsae031. doi: 10.1093/scan/nsae031 (PMC11223611; doi:10.1093/scan/nsae031)
Supplement: nsae031_Supp [file nsae031_supp.zip › scan-23-169-File002.docx]

**Supplementary Materials**

ROI ANOVA Interactions

For each ROI, no significant interactions were identified (see following table):

| **ROI** | **Interactions** | ***F* (*df*)** | ***p-value*** |
| --- | --- | --- | --- |
| Left Amygdala | Task*Gaming | .84 (1) | .37 |
|  | Valence*Gaming | .43 (1) | .51 |
|  | Task*Valence | .72 (1) | .40 |
|  | Task*Valence*Gaming | .00 (1) | .98 |
| Right Amygdala | Task*Gaming | .93 (1) | .34 |
|  | Valence*Gaming | .19 (1) | .67 |
|  | Task*Valence | 1.51 (1) | .23 |
|  | Task*Valence*Gaming | .19 (1) | .66 |
| Left Anterior Insula | Task*Gaming | .15 (1) | .70 |
|  | Valence*Gaming | .32 (1) | .57 |
|  | Task*Valence | .03 (1) | .87 |
|  | Task*Valence*Gaming | .003 (1) | .96 |
| Right Anterior Insula | Task*Gaming | .67 (1) | .42 |
|  | Valence*Gaming | .24 (1) | .63 |
|  | Task*Valence | 4.30 (1) | .05 |
|  | Task*Valence*Gaming | .40 (1) | .53 |
| Left IFG | Task*Gaming | .01 (1) | .97 |
|  | Valence*Gaming | .54 (1) | .47 |
|  | Task*Valence | .18 (1) | .67 |
|  | Task*Valence*Gaming | .01 (1) | .93 |
| Area of the Superior Temporal Cortex | Task*Gaming | .001 (1) | .98 |
|  | Valence*Gaming | .01 (1) | .95 |
|  | Task*Valence | .44 (1) | .51 |
|  | Task*Valence*Gaming | .08 (1) | .78 |
